# A Better Way

**Published:** 1875-06

**Authors:** 


A Better Way.—Some good mothers fly to the camphor or peppermint bottle on the slightest provocation. Camphor is the more dangerous drug, but both are capable of destroying life. As generally used, in the form of an alcoholic tincture, their potency is in no wise diminished, but rather increased by the addition of another poison.

Peppermint oil, from which the essence is made, is a powerful stimulant, and its capacity for harm is by no means inconsiderable. Ulceration of the stomach has been induced by it, and many diseases have followed its habitual use.

Camphor is a poisonous gum-resin, capable of readily inducing great nervous irritation. When taken in small doses it has much the effect of alcohol or opium. In large doses it occasions spasms and death. In any appreciable amount it irritates the mucus membrane of the stomach, and leads to constipation and ulceration. Even a few doses of this drug may lead to incurable dyspepsia. Yet thousands of families fly to the camphor-bottle for relief from every variety of pain.

It were a thousand times better that every camphor bottle in the land should be broken rather than that its contents should be indiscriminately employed. The potent drug ought never to be administered internally, except by a competent person, familiar with its power.

It would be a good thing if mothers could learn to depend upon water—cold, tepid or hot—to relieve a very large percentage of all bodily pain. There is nothing so innocent; nothing so effectual. Copious draughts of very hot water, aided, if need be, by hot water enemas, and hot water externally applied, will speedily relieve nine cases of colic in ten. Cold water is the most powerful local anæsthetic known. The pain of a sprained limb is quickly relieved by ice-cold water. The terrible pangs of whitlow or felon are cut short by intense heat. A large proportion of all cramps and spasms may be relieved by water of proper temperature and intelligently applied. We need a school for the education of doctors and nurses up to a moderate comprehension of the advantages of water, as an agent for the relief of every day aches and pains, whether slight or severe.

---

Young men and young women should be made to regard marriage as a sacred duty, and married life as the only true relation for social beings.

All honor and respect shonld be given to those who accept of love without wealth, and are willing to do and bear and suffer their part in the battle of life.

